# Supplementary material for: Unveiling the Bioactive Potential of the Invasive Jellyfish Phyllorhiza punctata Through Integrative Transcriptomic and Proteomic Analyses
Source: Biomolecules. 2025 Aug 4;15(8):1121. doi: 10.3390/biom15081121 (PMC12383608; doi:10.3390/biom15081121)
Supplement: Supplementary file 1 [file biomolecules-15-01121-s001.zip › Suplementary Material/FileS3/PEPFOLD4/XP_065054943_7525/clusters.html]

 PEP-FOLD clusters


Download archive of all models  here   

|  |  |  |
| --- | --- | --- |
| # | file | sOPEP |
| # cluster 1 - Size: 37 |  |  |
|  | XP\_065054943\_7525-model1 | -7.9746 |
|  | XP\_065054943\_7525-model1.1 | -7.9746 |
|  | XP\_065054943\_7525-model1.2 | -7.9746 |
|  | XP\_065054943\_7525-model1.3 | -7.9746 |
|  | XP\_065054943\_7525-model1.4 | -7.9746 |
|  | XP\_065054943\_7525-model1.5 | -7.9746 |
|  | XP\_065054943\_7525-model1.6 | -7.9746 |
|  | XP\_065054943\_7525-model1.7 | -7.9746 |
|  | XP\_065054943\_7525-model1.8 | -7.9746 |
|  | XP\_065054943\_7525-model1.9 | -7.89475 |
|  | XP\_065054943\_7525-model1.10 | -7.89475 |
|  | XP\_065054943\_7525-model1.11 | -7.89475 |
|  | XP\_065054943\_7525-model1.12 | -7.89475 |
|  | XP\_065054943\_7525-model1.13 | -7.89475 |
|  | XP\_065054943\_7525-model1.14 | -7.89475 |
|  | XP\_065054943\_7525-model1.15 | -7.89475 |
|  | XP\_065054943\_7525-model1.16 | -7.89475 |
|  | XP\_065054943\_7525-model1.17 | -7.89475 |
|  | XP\_065054943\_7525-model1.18 | -7.89475 |
|  | XP\_065054943\_7525-model1.19 | -7.89475 |
|  | XP\_065054943\_7525-model1.20 | -7.89475 |
|  | XP\_065054943\_7525-model1.21 | -7.89475 |
|  | XP\_065054943\_7525-model1.22 | -7.89475 |
|  | XP\_065054943\_7525-model1.23 | -7.89475 |
|  | XP\_065054943\_7525-model1.24 | -7.89475 |
|  | XP\_065054943\_7525-model1.25 | -7.89475 |
|  | XP\_065054943\_7525-model1.26 | -7.89475 |
|  | XP\_065054943\_7525-model1.27 | -7.89475 |
|  | XP\_065054943\_7525-model1.28 | -7.89475 |
|  | XP\_065054943\_7525-model1.29 | -7.89475 |
|  | XP\_065054943\_7525-model1.30 | -7.89475 |
|  | XP\_065054943\_7525-model1.31 | -7.89475 |
|  | XP\_065054943\_7525-model1.32 | -7.89475 |
|  | XP\_065054943\_7525-model1.33 | -7.83165 |
|  | XP\_065054943\_7525-model1.34 | -7.76996 |
|  | XP\_065054943\_7525-model1.35 | -7.65375 |
|  | XP\_065054943\_7525-model1.36 | -7.55926 |
| # cluster 2 - Size: 40 |  |  |
|  | XP\_065054943\_7525-model2 | -4.00227 |
|  | XP\_065054943\_7525-model2.1 | -4.00227 |
|  | XP\_065054943\_7525-model2.2 | -4.00227 |
|  | XP\_065054943\_7525-model2.3 | -4.00227 |
|  | XP\_065054943\_7525-model2.4 | -4.00227 |
|  | XP\_065054943\_7525-model2.5 | -4.00227 |
|  | XP\_065054943\_7525-model2.6 | -4.00227 |
|  | XP\_065054943\_7525-model2.7 | -4.00227 |
|  | XP\_065054943\_7525-model2.8 | -4.00227 |
|  | XP\_065054943\_7525-model2.9 | -4.00227 |
|  | XP\_065054943\_7525-model2.10 | -4.00227 |
|  | XP\_065054943\_7525-model2.11 | -4.00227 |
|  | XP\_065054943\_7525-model2.12 | -4.00227 |
|  | XP\_065054943\_7525-model2.13 | -4.00227 |
|  | XP\_065054943\_7525-model2.14 | -4.00227 |
|  | XP\_065054943\_7525-model2.15 | -4.00227 |
|  | XP\_065054943\_7525-model2.16 | -4.00227 |
|  | XP\_065054943\_7525-model2.17 | -3.93945 |
|  | XP\_065054943\_7525-model2.18 | -3.93945 |
|  | XP\_065054943\_7525-model2.19 | -3.93945 |
|  | XP\_065054943\_7525-model2.20 | -3.93945 |
|  | XP\_065054943\_7525-model2.21 | -3.93945 |
|  | XP\_065054943\_7525-model2.22 | -3.93945 |
|  | XP\_065054943\_7525-model2.23 | -3.93945 |
|  | XP\_065054943\_7525-model2.24 | -3.93945 |
|  | XP\_065054943\_7525-model2.25 | -3.93945 |
|  | XP\_065054943\_7525-model2.26 | -3.93945 |
|  | XP\_065054943\_7525-model2.27 | -3.93945 |
|  | XP\_065054943\_7525-model2.28 | -3.93945 |
|  | XP\_065054943\_7525-model2.29 | -3.76509 |
|  | XP\_065054943\_7525-model2.30 | -3.76509 |
|  | XP\_065054943\_7525-model2.31 | -3.76509 |
|  | XP\_065054943\_7525-model2.32 | -3.76497 |
|  | XP\_065054943\_7525-model2.33 | -3.68136 |
|  | XP\_065054943\_7525-model2.34 | -3.68136 |
|  | XP\_065054943\_7525-model2.35 | -3.66972 |
|  | XP\_065054943\_7525-model2.36 | -3.6574 |
|  | XP\_065054943\_7525-model2.37 | -3.6574 |
|  | XP\_065054943\_7525-model2.38 | -3.6574 |
|  | XP\_065054943\_7525-model2.39 | -3.49843 |
| # cluster 3 - Size: 23 |  |  |
|  | XP\_065054943\_7525-model3 | -3.90645 |
|  | XP\_065054943\_7525-model3.1 | -3.90645 |
|  | XP\_065054943\_7525-model3.2 | -3.84614 |
|  | XP\_065054943\_7525-model3.3 | -3.84614 |
|  | XP\_065054943\_7525-model3.4 | -3.84614 |
|  | XP\_065054943\_7525-model3.5 | -3.84614 |
|  | XP\_065054943\_7525-model3.6 | -3.84614 |
|  | XP\_065054943\_7525-model3.7 | -3.8131 |
|  | XP\_065054943\_7525-model3.8 | -3.8131 |
|  | XP\_065054943\_7525-model3.9 | -3.79432 |
|  | XP\_065054943\_7525-model3.10 | -3.79432 |
|  | XP\_065054943\_7525-model3.11 | -3.79432 |
|  | XP\_065054943\_7525-model3.12 | -3.74682 |
|  | XP\_065054943\_7525-model3.13 | -3.74682 |
|  | XP\_065054943\_7525-model3.14 | -3.74682 |
|  | XP\_065054943\_7525-model3.15 | -3.74682 |
|  | XP\_065054943\_7525-model3.16 | -3.68737 |
|  | XP\_065054943\_7525-model3.17 | -3.67715 |
|  | XP\_065054943\_7525-model3.18 | -3.67715 |
|  | XP\_065054943\_7525-model3.19 | -3.58966 |
|  | XP\_065054943\_7525-model3.20 | -3.58849 |
|  | XP\_065054943\_7525-model3.21 | -3.56206 |
|  | XP\_065054943\_7525-model3.22 | -3.56206 |
|  |  |  |
